# In Silico Molecular Study of Tryptophan Bitterness

**DOI:** 10.3390/molecules25204623

**Published:** 2020-10-11

**Authors:** Antonella Di Pizio, Alessandro Nicoli

**Affiliations:** Leibniz-Institute for Food Systems Biology at the Technical University of Munich, Lise-Meitner-Str. 34, 85354 Freising, Germany; a.nicoli.leibniz-lsb@tum.de

**Keywords:** TAS2Rs, GPCRs, amino acids, homology modeling, docking

## Abstract

Tryptophan is an essential amino acid, required for the production of serotonin. It is the most bitter amino acid and its bitterness was found to be mediated by the bitter taste receptor TAS2R4. Di-tryptophan has a different selectivity profile and was found to activate three bitter taste receptors, whereas tri-tryptophan activated five TAS2Rs. In this work, the selectivity/promiscuity profiles of the mono-to-tri-tryptophans were explored using molecular modeling simulations to provide new insights into the molecular recognition of the bitter tryptophan. Tryptophan epitopes were found in all five peptide-sensitive TAS2Rs and the best tryptophan epitope was identified and characterized at the core of the orthosteric binding site of TAS2R4.

## 1. Introduction

Tryptophan (three-letter code: Trp, one-letter code: W) is one of the nine essential, or indispensable, amino acids. It is required for the production of serotonin and therefore has an important role in many fundamental physiological functions such as sleep, mood, cognition, and behavior [1,2,3]. It is a key component in the human diet. Nutritional tryptophan-containing supplements proved to be highly beneficial in malnutrition and undernutrition states, derived often by certain pathological conditions [4]. Due to its role in the serotonin pathway, tryptophan levels are affected in the course of metabolic disorders, and tryptophan supplements are associated with the medical treatment of several diseases such as depression, sleep disorders, cognitive disorders, anxiety, or neurodegenerative diseases [5].

Among the free amino acids, tryptophan has the lowest bitter taste threshold (BTT: 4 mmol/L) [6]. The bitter taste of tryptophan can lead to a reduction in the consumption of tryptophan-based nutritional supplements [7]. To gain from their effect, patients must consume the prescribed amount of supplements for the recommended time, but sensory characteristics strongly affect the patients’ acceptability and the correct assumption of nutritional supplements [8].

In humans, the bitter taste is mediated by a repertoire of 25 bitter taste receptors (TAS2Rs) [9,10,11], representing a separate branch usually associated with class A G protein-coupled receptors (GPCRs) [12,13]. The recognition of bitter molecules is complex: bitter compounds can activate selectively one TAS2R or several receptors promiscuously [14].

Although essential branched amino acids trigger generally aversive bitter taste perception [6,15], very few studies focused on their molecular recognition by bitter taste receptors. The bitter taste of amino acids and peptides was found to be mediated by a pattern of five peptide-sensitive TAS2Rs (i.e., TAS2R1, -R4, -R14, -R39, -R46) [16]. Tryptophan (W) selectively activates, with limited potency, the bitter taste receptor TAS2R4, whereas di-tryptophan (WW) and tri-tryptophan (WWW) exhibit considerable increases in the potencies and recruitment of additional receptors, with the tri-tryptophan activating all peptide-sensitive TAS2Rs (Table 1) [16].

In this study, we used *in silico* methods to investigate the binding modes of this little series of tryptophan-containing peptides and then, by the comparison of the obtained poses, to gain insights into the molecular recognition of the bitter tryptophan. Understanding the molecular mechanisms leading to the unpleasant bitter taste of the tryptophan may lead to the development of new strategies to ensure the acceptability of tryptophan-containing supplements. Moreover, bitter taste receptors are also expressed in extra-oral tissues such as in the hearth, in the brain, and in the gastrointestinal tract [17]. Therefore, investigations of the interaction of TAS2Rs with amino acids and peptides can help shed light on the function of the ‘ecnomotopically’ [18] expressed TAS2Rs, whose endogenous ligands are not known yet, but peptides are definitely good candidates.

## 2. Results

Despite the recent advancements in GPCR structural biology, no TAS2R experimental structure is currently available and computational techniques are currently used to predict the binding modes of bitter compounds, both small molecules [19] and amino acids/peptides [20,21,22], into their cognate TAS2Rs. The orthosteric binding site of TAS2Rs was found to coincide with that of class A GPCRs and the multi-specificity of bitter compounds was achieved by using sub-pockets within the orthosteric binding site, allowing for different types of interactions for different ligands [12,23,24,25].

The five peptide-sensitive TAS2Rs (i.e., TAS2R1, -R4, -R14, -R39, -R46) share low sequence identity among them and this difference is even more pronounced in the binding site (Supplementary Material Figure S1). Here, structural models of all peptide-sensitive TAS2R binding sites were used to characterize the selectivity/promiscuity profiles of mono-to-tri-tryptophans and to identify the putative binding mode of tryptophan.

### 2.1. From the Promiscuous Tri-Tryptophan to the Selective Tryptophan

Tri-tryptophan is the most promiscuous of the analyzed compounds. According to the performed docking studies, it fills in the binding site of the three peptide-sensitive bitter taste receptors in a highly complementary manner, as quantified by Glide Standard Precision (SP) score values that were below −8 kcal/mol in all cases (Table 1). Docking scores in general [26], and Glide scores in particular [27,28], have been successfully used for capturing the main ligand-receptor interactions and comparing binding modes. Glide SP scores, used here as an approximation of the binding affinity, were in good agreement with the experimental data: lower binding energy (lower Glide SP score) was indeed observed for tri-tryptophan in complex with TAS2R4 (experimental EC_50_: 0.03 mM) than in complex with the other receptors (experimental threshold values of 0.1 mM) (Table 1).

**Figure 1:**
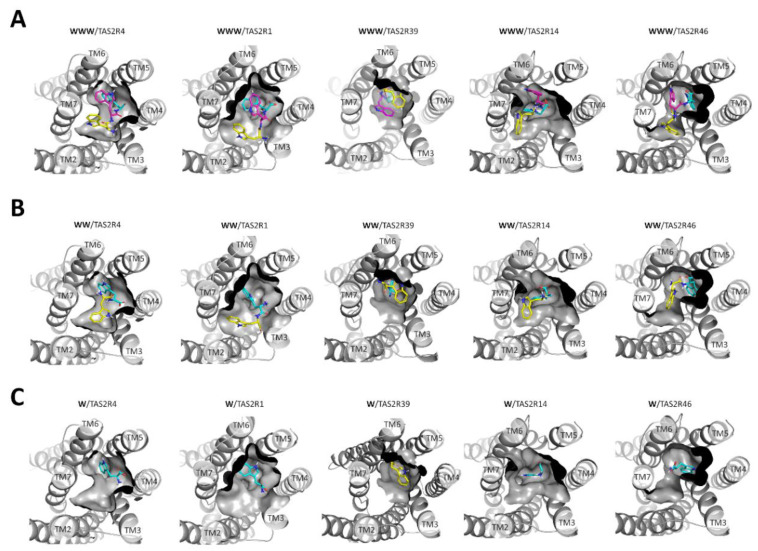
Predicted binding modes of tri-tryptophan (**A**), di-tryptophan (**B**), and tryptophan (**C**) into the binding sites of TAS2R4, -R1, -R39, -R14, and -R46. The receptors are shown in the same orientation in all representations. Tryptophan residues are colored in cyan when accommodated at the bottom of the orthosteric binding site, in magenta when close to the extracellular space, and in yellow when in between. Receptor sub-pockets are shown in Supplementary Material Figure S2.

To better understand the predicted affinity difference of the tri-tryptophan toward the different receptors, we analyzed the docking poses of the di-tryptophan, which resulted in activating TAS2R4, -R1, and -R39, but not TAS2R14 and -R46. Comparing the binding modes of tri-tryptophan and di-tryptophan, we noticed that the contribution of the third residue to the binding was different for the different receptors. Di-tryptophan (WW) and tri-tryptophan (WWW) had very similar Glide SP scores when docked into the binding sites of TAS2R1 and -R39, which suggests that the interaction of the third tryptophan residue (colored in magenta in Figure 1A) does not have a relevant contribution to the binding. Differently, within the TAS2R4 binding site, the third tryptophan residue was predicted to increase the affinity of the tri-tryptophan compared to the di-tryptophan of −2–29 kcal/mol. According to the docking analysis, only the tri-tryptophan, with its flexibility and bigger size, could ensure a good fitting within the binding sites of TAS2R14 and -R46, and the inactive di-tryptophan was predicted as a weak binder (Glide SP scores of −5.55 and −5.27 kcal/mol, respectively). Experimental data suggest that also the second tryptophan residue does not help gain potency toward TAS2R14 and -R46, since the single tryptophan is inactive toward these two receptors. In contrast, the second tryptophan residue results were determinant to elicit receptor activation for TAS2R1 and -R39.

Comparing the predicted binding modes of di-tryptophan (WW) and tryptophan (W), we could evaluate the contribution of the second tryptophan residue and therefore which of the sub-pockets identified for the tri- and di-tryptophans were the best tryptophan epitopes in each peptide-sensitive TAS2R (Figure 1C). The Glide SP score of the tryptophan into TAS2R14 and -R46 was high and comparable to that of the di-tryptophan. We also found low Glide SP scores for the tryptophan in complex with TAS2R1 and -R39, and this can be an explanation of the experimental results, which showed that one single tryptophan residue is not sufficient to lead to the receptor activation of TAS2R1 and -R39.

### 2.2. Predicted Binding Mode of Tryptophan into TAS2R4

**Figure 2:**
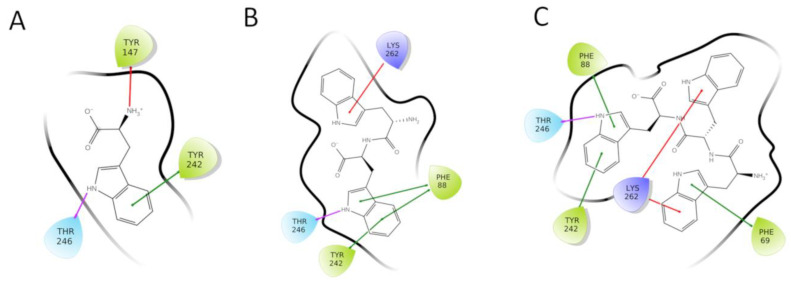
2D representations of the interactions established by tryptophan (**A**), di-tryptophan (**B**), and tri-tryptophan (**C**) within the TAS2R4 orthosteric binding site. H-bonds, π–π interactions, and π–cation interactions are represented as magenta, green, and red lines, respectively.

**Figure 3:**
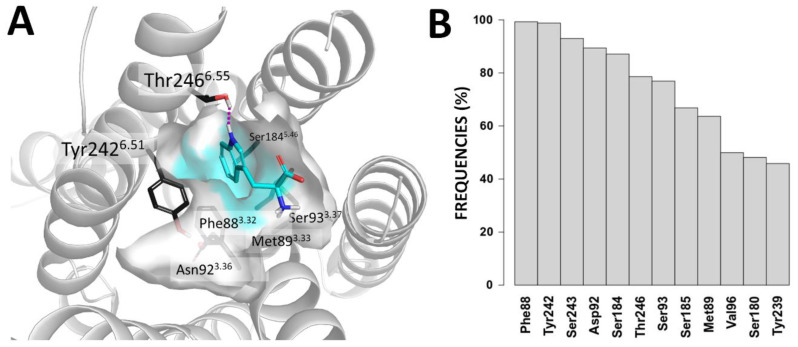
(**A**) Docking pose of the tryptophan within TAS2R4 epitope 1. The surface of the epitope is colored in cyan. Residues involved in shaping the epitope are shown as dark grey sticks. Tryptophan is represented as cyan sticks. The H-bond between the ligand and Thr246^6.55^ is shown as a magenta dashed line. (**B**) Frequencies of the contacts between the tryptophan with TAS2R4 residues during the MD simulation.

The docking results of the tri-tryptophan into the five peptide-sensitive TAS2Rs revealed the sub-pockets accommodating tryptophan residues in each receptor (all tryptophan epitopes are shown in Supplementary Material Figure S2). The binding of tryptophan residues to the individual epitopes had different affinities. Of all five peptide-sensitive receptors, only TAS2R4 could be activated by the tryptophan, and therefore this should contain the best tryptophan epitope. Among the docking poses of the tryptophan into the three identified TAS2R4 epitopes (Figure S2A), the highest score was obtained for a pose located at the core of the orthosteric binding site (epitope 1). The tryptophan was predicted to establish a π–π interaction with Tyr242^6.51^ and an H-bond with Thr246^6.55^ (Figure 2A and Figure 3A). Residue superscript numbering indicates Ballesteros–Weinstein (BW) positions and makes the comparison easier of the transmembrane positions with other TAS2Rs and class A GPCRs [13,29]. The interactions between the tryptophan and the residues Tyr242^6.51^ and Thr246^6.55^ were predicted to be maintained for di-tryptophan and tri-tryptophan, whereas the additional tryptophan residues were accommodated by other sub-pockets (Figure 1, Figures S2 and S3).

The resulting docking poses of tryptophan, di-tryptophan, and tri-tryptophan into TAS2R4 were submitted to post-docking Molecular Dynamics (MD) simulations of 20 ns. The root-mean-square deviation (RMSD) analyses indicated that, within the simulated time, the ligand positions did not deviate from the starting docking poses and, signally, the ligand indole moieties were very stable in their position within the identified TAS2R4 epitopes (Supplementary Material Figures S4 and S5).

In Figure 3, we report the 3D representation of the TAS2R4 epitope accommodating the tryptophan and the frequencies of contacts between tryptophan and TAS2R4, which were found during the post-docking MD simulation. Residues shaping this subpocket (Phe88^3.32^, Met89^3.33^, Asp92^3.36^, Ser93^3.37^, Ser184^5.46^, Tyr242^6.51^, Thr246^6.55^) were indeed among the residues found to be mostly involved in the binding process during the MD simulations.

## 3. Discussion

The tryptophan bitter taste was found to be mediated by TAS2R4 [16], but, to the best of our knowledge, the interaction of this relevant amino acid with the cognate bitter taste receptor has not been investigated. Interestingly, di-tryptophan and tri-tryptophan showed increased potency toward TAS2R4 and activate additional TAS2R members (Table 1). Therefore, tryptophan (W), di-tryptophan (WW), and tri-tryptophan (WWW) constitute an interesting case study to investigate structure–activity relationships into the bitter taste receptors and to characterize the molecular determinants leading to the tryptophan bitter taste.

We identified the sub-pockets accommodating the tryptophan in the orthosteric binding sites of the five peptide-sensitive bitter taste receptors and we found that these tryptophan epitopes are located in different regions of the receptors (Supplementary Material Figure S2). This also suggests that for peptides, as previously found for small molecules [12,23,24,25], the determinants for activation are receptor-specific. Moreover, the interaction of individual tryptophan residues with individual epitopes has a different contribution to the overall binding energy of the promiscuous tri-tryptophan, explaining the selectivity profile of the analyzed molecules against the five peptide-sensitive TAS2Rs.

Comparing the docking poses and Glide scores of the mono-to-tri-tryptophans, we unraveled the putative binding mode of tryptophan within the TAS2R4 binding site. The identified binding pose was highly scored (Glide SP score −7.05 kcal/mol) and resulted in being rather stable during the post-docking MD simulation. Tryptophan was surrounded by numerous hydrophobic/aromatic residues (Phe88^3.32^, Met89^3.33^, Tyr242^6.51^) and was predicted to interact with Tyr242^6.51^ and Thr246^6.55^ through π–π and H-bond interactions, respectively. Both BW positions 6.51 [23,24,30,31,32,33] and 6.55 [30,31,34] were previously indicated as agonist-interacting positions in other bitter taste receptors. Moreover, these interactions are indeed predicted to be maintained for di-tryptophan and tri-tryptophan (Figure 2), suggesting that the sub-pocket around Phe88^3.32^ and Tyr242^6.51^ is the best epitope for the recognition of tryptophan by TAS2R4 (TAS2R4 epitope 1, Figures S2A and S3A).

## 4. Materials and Methods

Homology modeling. Structural models of TAS2R1, -R14, and -R46 were generated in previous works [28,30,35]. The TAS2R14 receptor model was then used here as a template for modeling the initial structures of TAS2R4 and -R39. These structures were then refined by performing induced-fit simulations (Schrödinger Suite 2018-3 Induced Fit Docking protocol; Glide, Schrödinger, LLC, New York, NY, USA, 2016; Prime, Schrödinger, LLC, New York, NY, 2018) [36] with the most potent known ligand (i.e., TAS2R4 with quinine (EC_50_: 600 μM, activity threshold 10 μM [37,38]) and TAS2R39 with genistein (EC_50_: 50 μM [39]), predicted binding poses are shown in Supplementary Material, Figures S6 and S7). Docking was performed with the Glide Standard Precision (SP) accuracy level. The receptor conformation that gave the lowest Glide score was selected for the following docking studies.

Peptide docking. 3D structures of the analyzed amino acid and peptides were built with the “Build Peptide from Sequence” tool available in Maestro 11.7 and prepared for docking through the generation of protonation states at pH 7 ± 1.0 with LigPrep, as implemented in the Schrödinger Small-Molecule Drug Discovery Suite 2018-3 (Schrödinger, LLC, New York, NY, USA, 2018). Glide software (version 8.2, Schrödinger, LLC, New York, NY, USA, 2018) was used for docking the tryptophan, di-tryptophan, and tri-tryptophan to the peptide-sensitive TAS2R models. The grid box of all analyzed receptors was centroid of the main chains of the binding site residues (BW positions: 2.61, 3.29, 3.32, 3.36, 4.64, 5.35, 5.38, 5.39, 5.42, 5.46, 6.51, 6.55, and 7.39), using the option “Generate grid suitable for peptide docking” and allowing rotation of the binding site hydroxyl and thiol groups. The docking was then performed with the SP-peptide mode of Glide, which was specifically developed for the accurate prediction of binding complex geometries of peptides to receptors [40]. Docking poses of tri-tryptophan with the lowest Glide scores were then used as a selection filter for the docking poses of di-tryptophan and tri-tryptophan for each analyzed TAS2R: we considered the poses with the lowest scores among poses that reproduce the binding of one of the tripeptide residues. The Glide SP scores and ligand efficiency values of all selected poses are reported in Table 1 and Table S1, respectively. To provide additional score values for the identified binding modes, we applied the MM-GBSA scoring (Prime, Schrödinger, LLC, New York, NY, USA, 2018) of each of the predicted poses (Supplementary Material Table S2).

2D representations of the peptides/TAS2R4 binding modes (as reported in Figure 3 and Figure S3) were generated with the “Ligand Interaction Diagram” tool available in Maestro 11.7 (Schrödinger, LLC, New York, NY, USA, 2018).

Post-docking Molecular Dynamics (MD) simulations. The coordinates of the analyzed ligands in complex with TAS2R4 were embedded into a 1-palmitoyl-2oleyl-sn-glycerol-3-phosphocholine (POPC) bilayer of 90 × 90 Å using VMD Membrane Builder Plugin Tool [41]. The orientation of the TAS2R4 homology model within the membrane bilayer was obtained from the coordinates of the β2 adrenergic receptor (PDB ID: 3SN6), as deposited in the Orientations of Proteins in Membranes (OPM) database [42]. Overlapping lipids (within 0.55 Å) were removed upon protein insertion, and the systems were solvated with TIP3P water molecules at 15 Å from protein atoms using VMD Solvate plugin 1.5 and neutralized by Na^+^/Cl^−^ to reach a final physiological concentration of 0.154 M by using VMD Autonize plugin 1.3 [41,43].

All MD simulations with periodic boundary conditions were carried out with ACEMD (Acellera, version 3.2) [44] using the CHARMM36 force field [45,46]. The systems were equilibrated through a 2000 conjugate gradient step minimization, followed by 20 ns of MD simulation in the NPT ensemble by applying initial constrains that were gradually reduced (positional constraints of 5 kcal·mol^−1^·Å^−2^ on ligand, protein, and lipid phosphor atoms in the first 5 ns, positional constraints of 5 kcal·mol^−1^·Å^−2^ on ligand and protein atoms in the following 10 ns, positional constraints of 5 kcal·mol^−1^·Å^−2^ on ligand atoms and protein Cα atoms in the last 5 ns). During the equilibration, the temperature was maintained at 310 K using a Langevin thermostat with a low damping constant of 1 ps^−1^, and the pressure was maintained at 1 atm using a Berendensen barostat. The M-SHAKE algorithm [47,48] was used to constrain the bond lengths involving hydrogen atoms. The equilibrated systems were then subjected to 20 ns of the unrestrained MD simulation run (NVT ensemble, timestep  =  2 fs, T = 310 K, damping constant  =  0.1 ps^−1^). Long-range Coulombic interactions were handled using the particle mesh Ewald summation method (PME) [49] with a grid size rounded to the approximate integer value of the cell wall dimensions. A non-bonded cutoff distance of 9 Å with a switching distance of 7.5 Å was used.

The number of contacts between the analyzed ligands (W, WW, WWW) and TAS2R4 was computed using the contactFreq.tcl script in VMD [41], setting a distance cut-off of 4 Å. Root-mean-square deviations (RMSDs) to the docking poses were computed for ligand heavy atoms and ligand indole scaffold heavy atoms using ProDy [50]. The resulting interaction frequencies were plotted using Bar Plots in R.

## 5. Conclusions

Tryptophan is an essential amino acid, required for the production of serotonin. It is the most bitter amino acid and its bitterness was found to be mediated by the bitter taste receptor TAS2R4 [16]. In this work, we analyzed the putative interaction of tryptophan, di-tryptophan and try-tryptophan into the cognate bitter taste receptors. Combining comparative modeling, docking, and MD investigations allowed us (i) to rationalize the factors contributing to improving the activity values toward individual TAS2Rs while growing the peptide chain; (ii) to investigate the selectivity/promiscuity profile of the five peptide-sensitive TAS2Rs toward analyzed tryptophan peptides; and (iii) to identify and characterize the best tryptophan epitope.

## Figures and Tables

**Table 1:** Glide SP scores (kcal/mol) of mono-to-tri-tryptophans docked into peptide-sensitive TAS2Rs. Activity values (threshold and EC_50_ when available) of TAS2Rs activated by mono-to-tri-tryptophans, as determined by Kohl et al. 2013 [16]. Cells are colored in green when the peptide was found to be active with the calcium-imaging assay and in red if potency could not be determined.

		TAS2R4	TAS2R1	TAS2R39	TAS2R14	TAS2R46
**WWW**	Activity (mM)	0.01 (EC_50_: 0.03 ± 0.005)	0.1	0.1	0.1	0.1
	Glide score	−10.88	−8.82	−8.10	−8.48	−8.20
**WW**	Activity (mM)	1.0 (EC_50_: 0.66 ± 0.03)	0.3	1.0		
	Glide score	−8.59	−8.71	−8.35	−5.55	−5.27
**W**	Activity (mM)	10				
	Glide score	−7.05	−5.36	−5.34	−5.43	−5.05

## Supplementary Materials

Supplementary materials are available online, Figure S1: Sequence alignment of analyzed TAS2Rs, Figure S2. Tryptophan epitopes in the orthosteric binding sites of TAS2R4, TAS2R1, TAS2R39, TAS2R14, TAS2R46. Figure S3. 2D representations of the interactions established by tryptophan, di-tryptophan, and tri-tryptophan within the orthosteric binding site of TAS2R1, TAS2R39, TAS2R14, TAS2R46. Figure S4. RMSD plots of ligand heavy atoms through 20ns MD of tryptophan/TAS2R4, di-tryptophan/TAS2R4, and tri-tryptophan/TAS2R4. Figure S5. RMSD plots of ligands’ indole moieties through 20 ns MD. Figure S6. Predicted binding mode of quinine within the TAS2R4 orthosteric binding site. Figure S7. Predicted binding mode of quinine within the TAS2R39 orthosteric binding site. Table S1: Ligand Efficiency (LE) values, as calculated by docking simulations of mono-to-tri-tryptophans towards peptide-sensitive TAS2Rs. Table S2: MM-GBSA dG bind values calculated with Prime.
