# Supplementary material for: In Silico Molecular Study of Tryptophan Bitterness
Source: Molecules. 2020 Oct 11;25(20):4623. doi: 10.3390/molecules25204623 (PMC7587216; doi:10.3390/molecules25204623)
Supplement: Supplementary file 1 [file molecules-25-04623-s001.pdf]

molecules

MDPI

Supplementary Material:

In Silico Molecular Study of Tryptophan Bitterness

Antonella Di Pizio * and Alessandro Nicoli

Leibniz-Institute for Food Systems Biology at the Technical University of Munich, Lise-Meitner-Str. 34, 85354 Freising, Germany; a.nicoli.leibniz-lsb@tum.de

* Correspondence: a.dipizio.leibniz-lsb@tum.de; Tel.: +49-816-171-6516

Figure S1. Sequence alignment of analyzed TAS2Rs. (**A**) Sequence alignment of TAS2R1 (residues 1-291), −4 (1–294), −14 (1–294), −39 (29–325) and −46 (1–293) colored by alignment quality and residue type, and annotated with x.50 BW numbers and TM binding site residues (as green stars). (**B**) Sequence identity matrix. (**C**) Sequence identity matrix of the binding site.

A

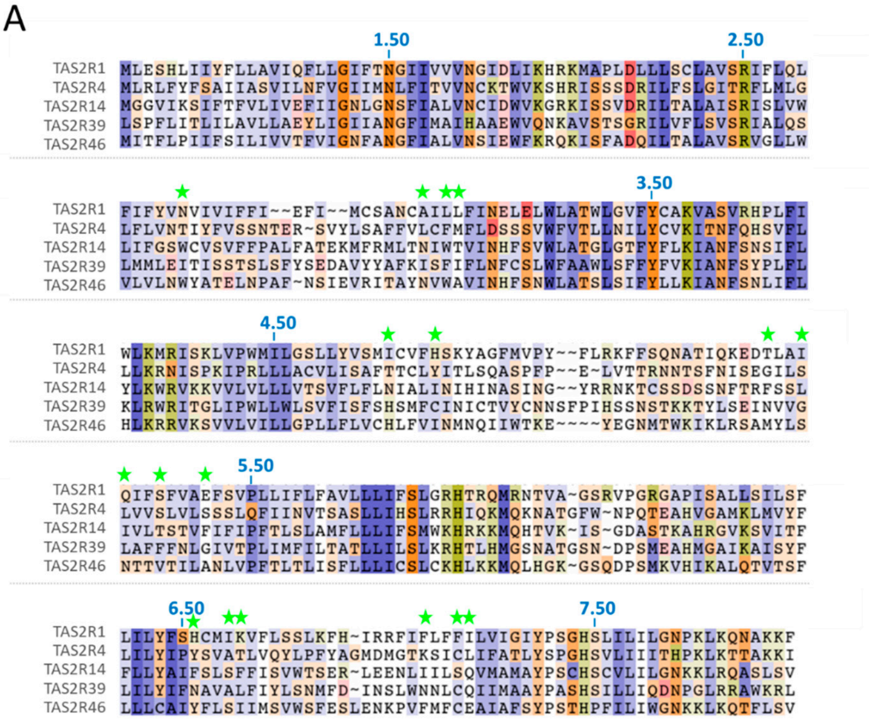

B

	TAS2R14	TAS2R46	TAS2R1	TAS2R4	TAS2R39
TAS2R14	100	42	22	24	24
TAS2R46	42	100	22	24	25
TAS2R1	22	22	100	20	23
TAS2R4	24	24	20	100	28
TAS2R39	24	25	23	28	100

C

	TAS2R14	TAS2R46	TAS2R1	TAS2R4	TAS2R39
TAS2R14	100	31	8	0	15
TAS2R46	31	100	8	15	8
TAS2R1	8	8	100	8	8
TAS2R4	0	15	8	100	15
TAS2R39	15	8	8	15	100

Figure S2. Tryptophan epitopes in the orthosteric binding sites of TAS2R4 (**A**), TAS2R1 (**B**), TAS2R39 (**C**), TAS2R14 (**D**), TAS2R46 (**E**).

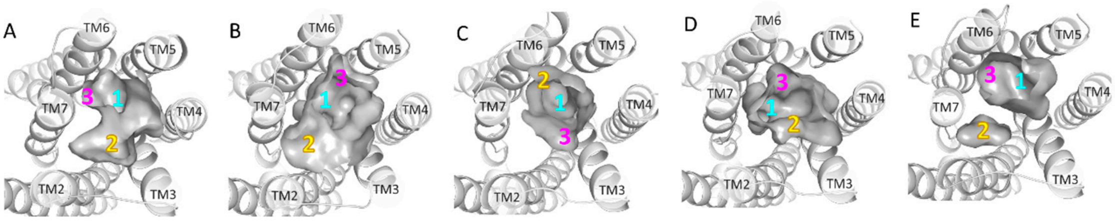

Main epitope defining residues:

<u>TAS2R4:</u> Phe88 and Tyr242 in epitope 1, Lys262 in epitope 2, Phe69 in epitope 3

<u>TAS2R1:</u> Leu86 and Phe262 in epitope 1, Val69 in epitope 2, Phe252 in epitope 3

<u>TAS2R39:</u> Phe122 and Leu125 in epitope 1, Tyr281 in epitope 2, Tyr110 and Phe117 in epitope 3

<u>TAS2R14:</u> Trp89 and Phe247 in epitope 1, Trp66 in epitope 2, Phe171 in epitope 3

<u>TAS2R46:</u> Tyr85 and Trp88 in epitope 1, Val 179 in epitope 2, Tyr241 and Ile245 in epitope 3

Figure S3. 2D representations of the interactions established by tryptophan, di-tryptophan, and tri-tryptophan within the orthosteric binding site of TAS2R1 (**A**), TAS2R39 (**B**), TAS2R14 (**C**), TAS2R46 (**D**). H-bonds, π-π interactions and π-cation interactions are represented as magenta, green and red lines, respectively.

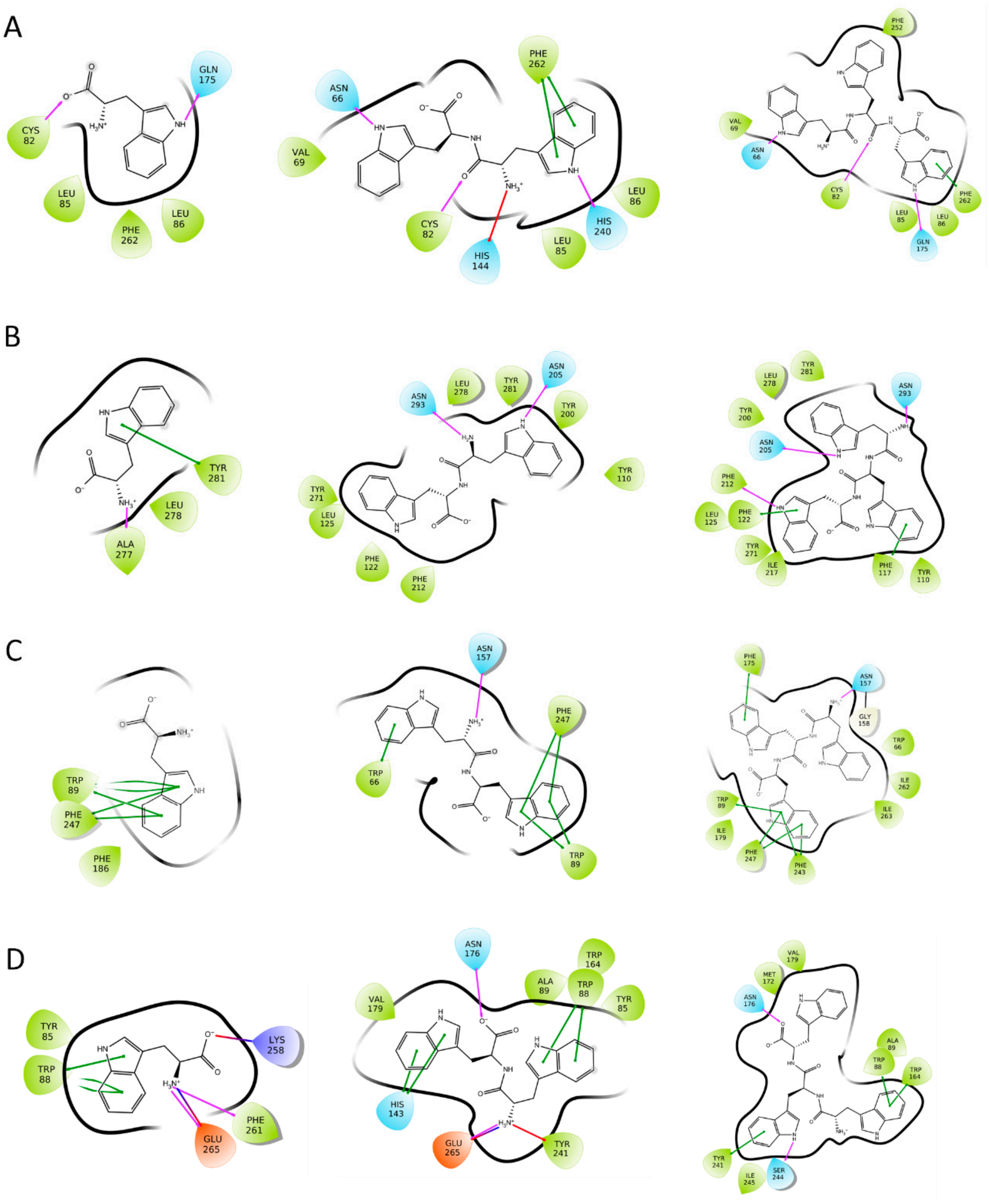

Figure S4. RMSD plots of ligand heavy atoms through 20ns MD of tryptophan/TAS2R4 (**A**), di-tryptophan/TAS2R4 (**B**) and tri-tryptophan/TAS2R4 (**C**).

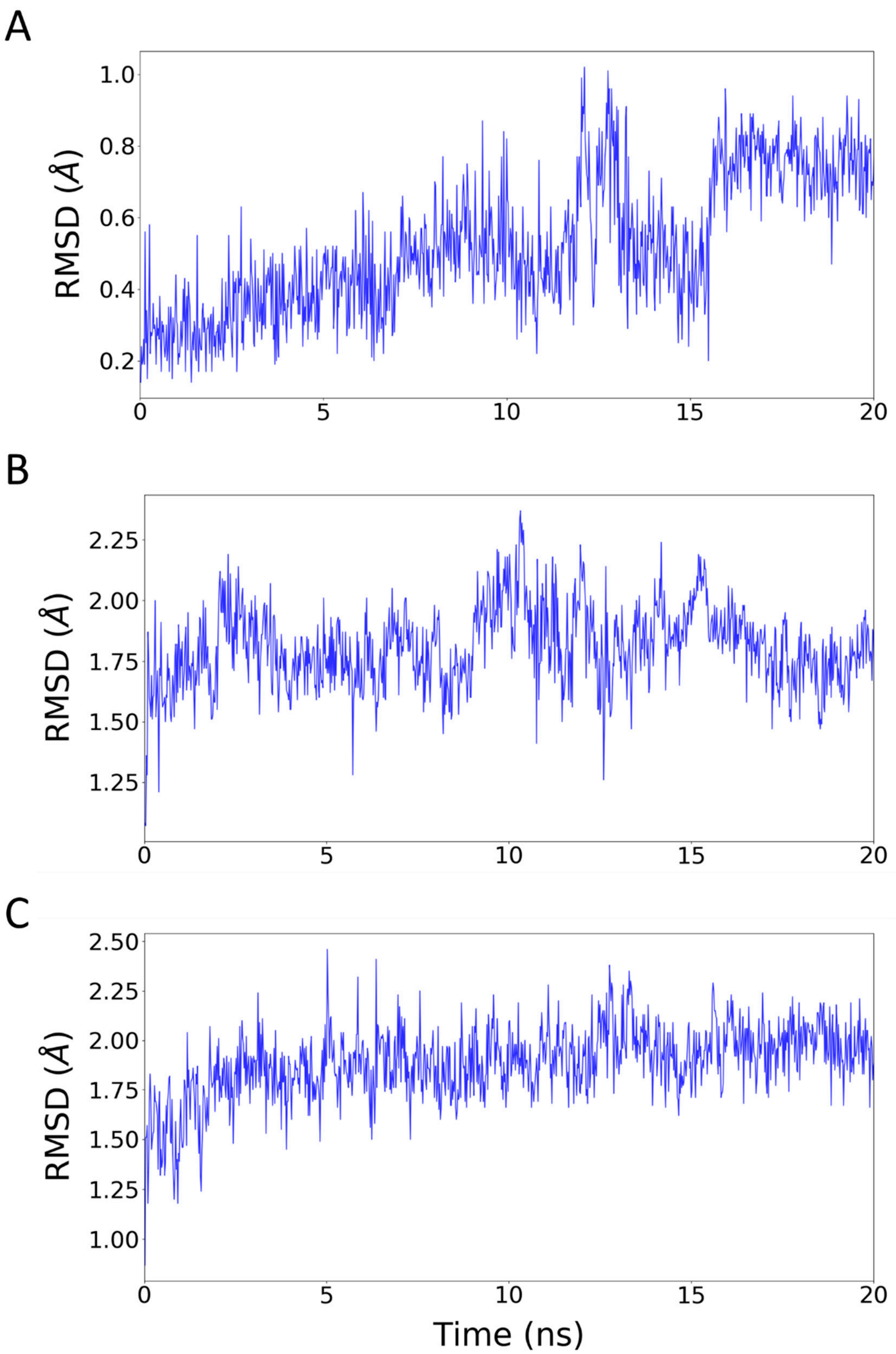

Figure S5. RMSD plots of ligands' indole moieties through 20 ns MD. (**A**) Indole moiety of tryptophan; (**B**) indole moieties of di-tryptophan binding to epitope 1 (cyan) and epitope 2 (yellow); (**C**) indole moieties of the tri-tryptophan binding to epitope 1 (cyan), epitope 2 (yellow), and epitope 3 (magenta).

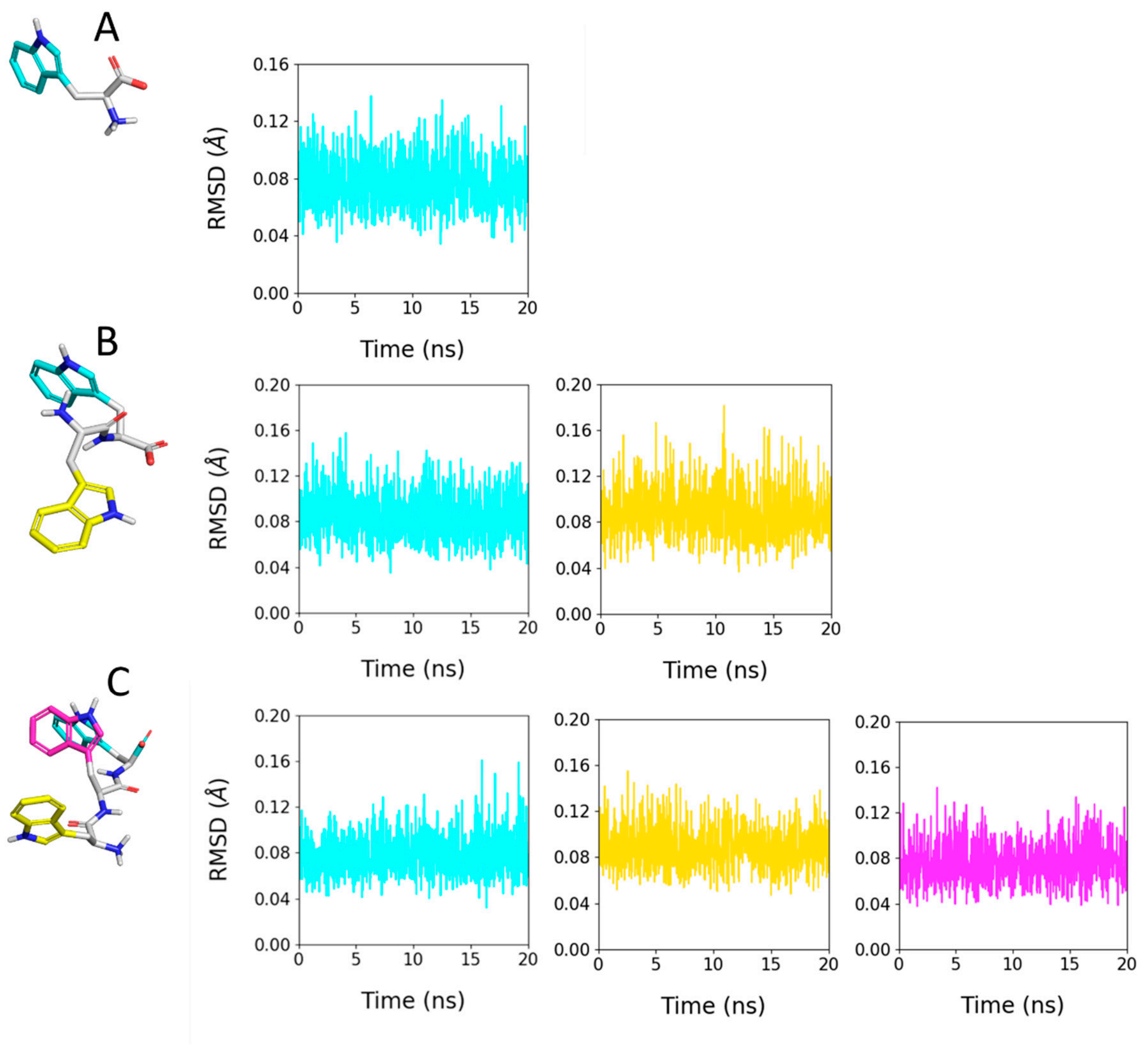

Figure S6. Predicted binding mode of quinine within the TAS2R4 orthosteric binding site. Quinine forms cation-π interaction with F88$^{3.32}$, π-π interactions with F88$^{3.32}$ and Y242$^{6.51}$, hydrophobic interactions with V85$^{3.29}$ and Y147$^{4.64}$, H-bond interaction with T246$^{6.55}$ (Glide SP score: -8.55 kcal/mol). Y147$^{4.64}$ was suggested as a TAS2R4 point contact for quinine recognition also in other studies since Y147A impair TAS2R4 activation by quinine (Pydi, Sobotkiewicz et al. 2014).

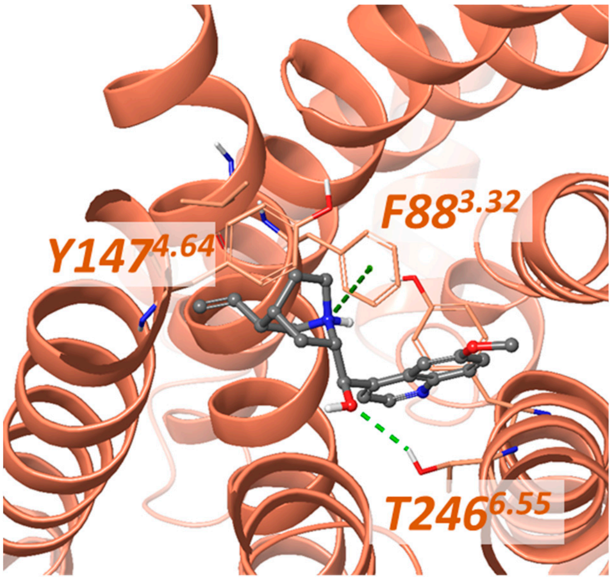

Figure S7. Predicted binding mode of quinine within the TAS2R39 orthosteric binding site. Genistein interacts with F117$^{3.32}$ and F212$^{5.42}$ through π-π stacking interactions, with K114$^{3.29}$ through cation-π interaction, and with N121$^{3.36}$ through an H-bond interaction (Glide score: −8.34 kcal/mol). Ligand-receptor interactions are shown as green dashed lines.

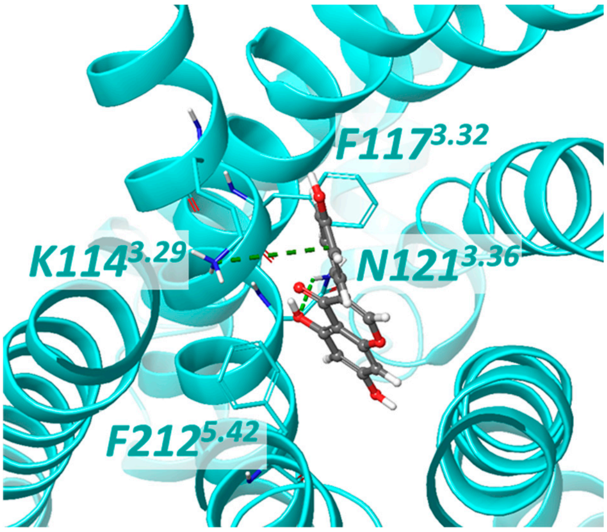

Table S1. Ligand Efficiency (LE) values, as calculated by docking simulations of mono-to-tri-tryptophans towards peptide-sensitive TAS2Rs. Cells are colored in green when the peptide was found to be active with calcium-imaging assay and in red if potency could not be determined (Kohl, Behrens et al. 2013).

	TAS2R4	TAS2R1	TAS2R39	TAS2R14	TAS2R46
**W**	−0.46	−0.36	−0.37	−0.36	−0.34
**WW**	−0.27	−0.30	−0.26	−0.19	−0.15
**WWW**	−0.25	−0.20	−0.19	−0.19	−0.18

Table S2. MM-GBSA dG bind values calculated with Prime (Schrödinger, LLC, New York, NY, USA, 2018). Cells are colored in green when the peptide was found to be active with calcium-imaging assay and in red if potency could not be determined (Kohl, Behrens et al. 2013).

	TAS2R4	TAS2R1	TAS2R39	TAS2R14	TAS2R46
**W**	−35.50	−23.55	−27.38	−39.64	−38.38
**WW**	−36.25	−41.42	−74.25	−50.96	−33.23
**WWW**	−64.73	−47.28	−84.76	−71.58	−72.25

References:

Kohl, S., M. Behrens, A. Dunkel, T. Hofmann and W. Meyerhof (2013). "Amino Acids and Peptides Activate at Least Five Members of the Human Bitter Taste Receptor Family." Journal of Agricultural and Food Chemistry **61**(1): 53-60.

Pydi, S. P., T. Sobotkiewicz, R. Billakanti, R. P. Bhullar, M. C. Loewen and P. Chelikani (2014). "Amino Acid Derivatives as Bitter Taste Receptor (T2R) Blockers." Journal of Biological Chemistry **289**(36): 25054-25066.
